# Theta transcranial alternating current stimulation over the prefrontal cortex enhances theta power and working memory performance

**DOI:** 10.3389/fpsyt.2024.1493675

**Published:** 2025-01-14

**Authors:** Ranjan Debnath, Osama Elyamany, Jona Ruben Iffland, Jonas Rauh, Michael Siebert, Elisa Andraes, Gregor Leicht, Christoph Mulert

**Affiliations:** ^1^ Centre for Psychiatry and Psychotherapy, Justus-Liebig University Giessen, Giessen, Germany; ^2^ Centre for Mind, Brain and Behaviour (CMBB), University of Marburg and Justus-Liebig University Giessen, Marburg, Germany; ^3^ Department of Psychiatry and Psychotherapy, University Medical Centre Hamburg-Eppendorf, Hamburg, Germany

**Keywords:** transcranial alternating current stimulation, EEG, working memory, theta power, dorsolateral prefrontal cortex

## Abstract

**Introduction:**

Transcranial alternating current stimulation (tACS) is a promising tool for modulating brain oscillations. This study investigated whether 5 Hz tACS could modulate neural oscillations in the prefrontal cortex and how this modulation impacts performance in working memory (WM) tasks.

**Method:**

In two sessions, 28 healthy participants received 5 Hz tACS or sham stimulation over the left dorsolateral prefrontal cortex (DLPFC) while performing tasks with high and low WM loads. Resting-state EEG was recorded before and after stimulations for 5 minutes. EEG power was measured at electrodes surrounding the stimulation site.

**Results:**

The results showed that tACS significantly improved reaction time (RT) compared to sham stimulation. This effect was task-specific, as tACS improved RT for hit responses only in high WM load trials, with no impact on low-load trials. Moreover, tACS significantly increased EEG power at 5 Hz and in the theta band compared to pre-stimulation levels.

**Discussion:**

These findings demonstrate that tACS applied over left DLPFC modulates post-stimulation brain oscillations at the stimulation sites – known as tACS after-effects. Furthermore, the results suggest that 5 Hz tACS enhances response speed by elevating task-related activity in the prefrontal cortex to an optimal level for task performance.

**Conclusion:**

In summary, the findings highlight the potential of tACS as a technique for modulating specific brain oscillations, with implications for research and therapeutic interventions.

## 1 Introduction

Working memory (WM) is a complex cognitive process that temporarily stores and manipulates information required for various cognitive functions including language comprehension, learning, problem-solving, and decision-making (1, 2). Deficits in WM leading to diminished cognitive activities have been observed in several neuropsychiatric and neurological disorders, such as depression (3, 4), schizophrenia (5–7), ADHD (8, 9), and Alzheimer’s disease (10, 11). Neuroimaging studies have identified brain regions, including the prefrontal cortex, superior and inferior parietal lobules, and the inferior temporal cortex, that underlie different components of WM (12–15). Moreover, electroencephalography (EEG) and magnetoencephalography (MEG) studies have demonstrated that neural oscillations within these brain regions play a major role in facilitating WM processing. WM is associated with enhanced oscillations and neural synchrony across multiple frequencies (16–19). However, there is evidence that specific frequency bands are particularly relevant to aspects of working memory. Notably, theta oscillations in the dorsolateral prefrontal cortex (DLPFC) have been found to be associated with WM performance (20–22). Alekseichuk et al. (16) found that synchronized theta and high gamma oscillations in the prefrontal cortex are essential for efficient spatial working memory function, particularly under higher cognitive demands. Theta appears to act as a coordinating rhythm and a potential organizer of working memory processing (16, 23).

The association between DLPFC theta oscillations and WM has been investigated by attempting to modulate oscillations within DLPFC using non-invasive brain stimulation techniques. Among brain stimulation techniques, transcranial alternating current stimulation (tACS) emerges as a promising tool in research and therapeutic endeavors due to its ability to modulate cortical excitability using low-intensity electrical currents (24). tACS delivers alternating electric current to specific brain regions through scalp electrodes affecting the neural oscillations at a particular frequency (25–28). tACS is believed to interact with ongoing rhythmic cortical activities and directly influence cognitive processes by entraining underlying neural oscillations at the frequency of stimulation (29, 30). The ability of tACS to modulate endogenous oscillations at stimulated frequency allows more direct and selective enhancements of processes underlying cognitions. Indeed, numerous studies reported that tACS enhanced performance in working memory (31–33), learning (34, 35), and decision-making (36, 37). Moreover, selective entrainment of specific neural oscillations through tACS has significant clinical implications as it could aid individuals with psychiatric and neurological disorders. Recent clinical applications of tACS have shown encouraging results in alleviating negative symptoms in several psychiatric conditions such as depression (38–40) and schizophrenia (28, 41).

Previous studies investigating the effects of tACS over DLPFC on working memory have produced mixed results (for details review Booth et al. (42); Senkowski et al. (43)). Several studies have reported that tACS applied simultaneously over frontal and parietal areas significantly improved WM performance in healthy individuals (18, 19, 30, 31, 44–47). However, studies examining the effects of tACS applied solely or separately over brain areas overlying the DLPFC are limited and the findings are inconsistent. For instance, Jaušovec et al. (45) observed positive effects of theta tACS on WM performance across different paradigms of WM when delivered over the frontal or parietal cortex. Jaušovec and Jaušovec (31) found that theta tACS over the left parietal brain area significantly increased WM capacity compared to sham tACS; however, no such effect was observed for tACS over the left frontal region. Our previous study found that 5 Hz tACS over the left DLPFC significantly improved performance in high-load WM tasks whereas tACS did not affect performance in low-load tasks (Rauh et al. (33)). These varying findings highlight the complexity of tACS effects on working memory and suggest that its impact may depend on multiple factors. Further research is needed to better understand the conditions under which tACS might influence WM performance and cognitive function.

Besides the effects of tACS on cognitive functions, a key area of research in tACS is its after-effects - lasting changes in brain activity and cognitive performance after the stimulation ends. Among the various frequencies used in tACS, theta and alpha frequencies have garnered particular interest due to their association with working memory, attention, and other cognitive functions. However, the existence and nature of tACS after-effects are debated, with studies reporting both positive and null results. For instance, Zaehle et al. (30) and Vossen et al. (29) found that alpha tACS improved cognitive performance and significantly increased alpha power compared to sham stimulation even 30 minutes after the stimulation ended. In contrast, Lafleur et al. (48) found no after-effects following 10 Hz and 20 Hz tACS applied over sensorimotor regions. Although many studies have explored tACS after-effects at alpha frequency (for a detailed review see De Koninck et al. (49)), research on tACS after-effects at theta frequency is scarce. D’Atri et al. (50) applied 5 Hz tACS to bilateral frontotemporal areas and observed significant increases in theta power in the parietooccipital area compared to the sham stimulation. Conversely, Pahor and Jaušovec (32) found a decrease in theta power measured 25 minutes after theta tACS stimulation. Briley et al. (51) reported increased task-related frontal theta power following theta tACS but observed no significant differences in resting theta power at 5 and 12 minutes post-stimulation compared to sham. These disparate findings highlight the need for further research to explore how to achieve sustained after-effects with theta tACS.

The present study investigates how targeted modulation of oscillations in the DLPFC influences task performance in healthy individuals. Building on our previous findings (Rauh et al. (33)), we applied 5 Hz (theta) tACS over the left DLPFC and examined its effects on performance in WM tasks. We hypothesized that theta tACS applied to the DLPFC would enhance performance in WM tasks compared to sham stimulation. Additionally, we explored whether theta tACS induces lasting changes in neural oscillations beyond the stimulation period - referred to as tACS after-effects. To this end, resting-state EEG was collected before and after the stimulations, and spectral power was measured from the EEG data. We anticipated an increase in post-stimulation theta power compared to pre-stimulation (baseline) levels. Furthermore, we investigated whether this enhancement in power was specific to the frequency of tACS.

## 2 Materials and methods

### 2.1 Participants

Twenty-eight healthy individuals participated in this study (Male = 10, Mean age = 32.5, SD = 13.22). Individuals with a history of neurological or psychiatric disease were excluded. All had normal or corrected-to-normal vision. They received compensation for their participation in the study. Each participant gave written informed consent to participate in the study. The Ethics Committee of the Justus-Liebig University Giessen reviewed and approved the study protocol.

### 2.2 Working memory tasks

The delayed match-to-sample task that examines load effects in visual WM was used in this study (33). The stimuli consisted of non-natural visual objects, specifically blurred outlines of random Tetris shapes (BORTs), presented under two conditions with varying WM loads. BORTs were chosen for their novelty and difficulty in verbalizing. The high-load condition featured four visual objects, while the low-load condition included two. To prevent recognition of previously viewed stimuli and subsequent ceiling effects, a large set of 504 BORTs was generated using a custom MATLAB script (448 for the experimental session and 56 for training). Within each session, no stimulus was repeated except for matching probe stimuli.

### 2.3 Procedure

**Figure 1:**
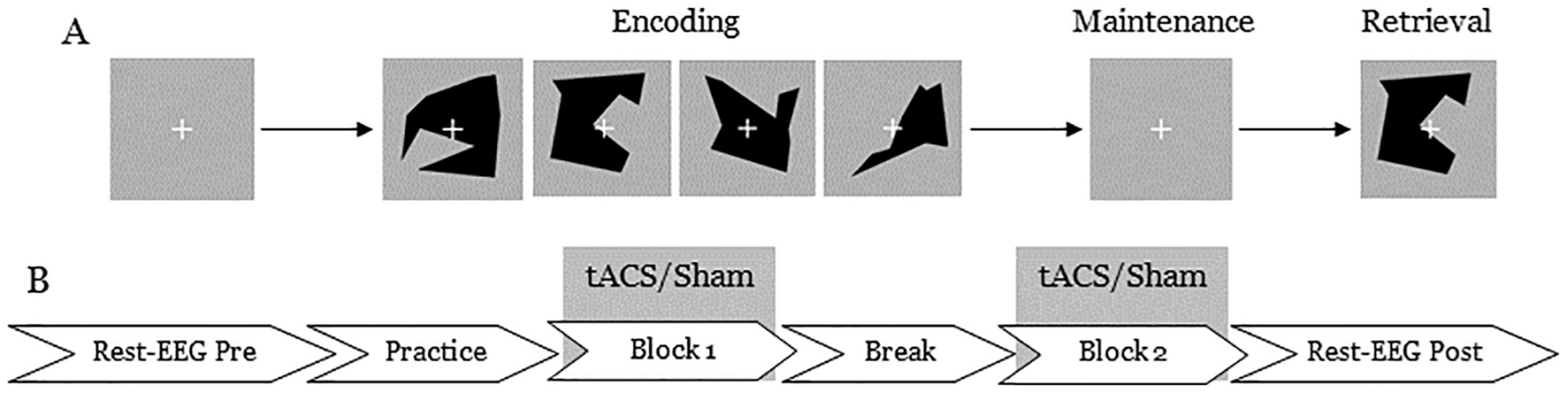
An illustration of **(A)** the delayed match-to-sample task used in the experiment and **(B)** stimulation and EEG data recording protocol. **(A)** Each trial began with a fixation cross for 2 seconds, followed by either 2 (low-load) or 4 (high-load) visual objects. Then, another fixation cross was presented for 2 seconds, followed by a probe stimulus. Participants responded by pressing a button to indicate whether they had seen the probe stimulus earlier. Each block lasted approximately 20 minutes. **(B)** Resting state EEG was collected for 5 minutes before and after the stimulation.

The experiment was conducted in an electrically shielded and acoustically attenuated cabin. Participants were seated at a table, and the stimulus was presented using Presentation software (Neurobehavioral Systems, Berkeley, United States). They performed the delayed match-to-sample task described above. Each trial consisted of three phases: encoding, maintenance, and retrieval (**Figure 1**). During the encoding phase, either two (low-load condition) or four (high-load condition) different visual objects were shown sequentially for 600 ms each, resulting in an encoding phase duration of 1.2 to 2.4 s depending on the condition. Following the encoding phase, a fixation cross was displayed for 2 s, during which participants were instructed to memorize the displayed items (maintenance phase). In the retrieval phase, a probe stimulus was presented for 2 s, and participants were asked to indicate as quickly and accurately as possible whether this probe stimulus had been shown during the encoding phase. Responses were made via button press with the left index finger for a mismatch and the right index finger for a match. The intertrial interval was set to 3.5 s. The position of the target stimulus (first, second, third, or fourth in the encoding phase) was equally distributed and remained constant within each trial set. Each experimental session consisted of two blocks of 80 trials (40 trials per condition), presented in a randomized order. At the beginning of each experimental session, a training session of 16 trials was conducted to familiarize participants with the task. This study employed a cross-over design in which each subject participated in both sham and theta tACS - spaced 7 days apart. The same procedure was repeated for both sham and theta sessions. The order of the sham and tACS sessions was pseudo-randomized and balanced across participants. Each experimental session lasted approximately 50 minutes.

### 2.4 tACS parameters

A DC Stimulator MC (neuroConn GmbH, Ilmenau, Germany) was used for the electrical stimulation. The stimulation was delivered to the scalp area overlying the left DLPFC at 5 Hz. The 5 Hz stimulation frequency was chosen because, as discussed above, the effects of tACS at a slow theta frequency on brain rhythms have not been extensively studied. Moreover, 5 Hz oscillation has been used as an approximation of theta activity in previous studies employing the visual delayed match-to-sample paradigm, which is comparable to the task used in the present study (33, 52). We targeted the left DLPFC at coordinates (x, y, z = −40, 37, 24) in MNI space, identified in a previous study by Rauh et al. (33). Consequently, we deployed a “left frontal” high-definition model comprising three return electrodes positioned at F1, FC5 and AF3, and one stimulating electrode at F3. The anodal rubber electrode had a diameter of 2 cm and was surrounded by 3 “cathodal” Ag/AgCl electrodes. First, an EEG cap was placed on the participant’s head, and the target position for the rubber electrode was marked with a pen. The cap was then removed, and the rubber electrode was attached to the marked position using Ten20 paste (Weaver and Company, Aurora, USA). Subsequently, the EEG cap was refitted onto the head, with the remaining stimulation electrodes integrated directly into the cap. To minimize impedance, Signagel^®^ Electrode Gel (Parker Laboratories, Fairfield, USA) was applied. To achieve a stimulation current of 1.5 mA (peak-to-peak for tACS) at F3, the stimulation currents were weighted -690 μA at F1 and -405 μA at both FC5 and AF3. The 5 Hz stimulation included a 10-second ramp-up and a 10-second ramp-down period. For the sham condition, the stimulation protocol included a 10-second ramp-up, 10 seconds of stimulation, and a 30-second ramp-down. In the sham stimulation, the current was turned off after the ramp-down period and remained off for the rest of the stimulation period. The stimulation session lasted 21 minutes.

### 2.5 EEG data recording and preprocessing

EEG data were recorded with 64 Ag/AgCl electrodes mounted on an elastic cap (ActiCaps, Brain Products, Munich, Germany), using the Brain Vision Recorder software version 1.21.0303 (Brain Products, Munich, Germany). The TP10 electrode was used as reference and the EEG data were sampled at 5000 Hz. Impedances were kept below 10 kΩ. EEG data were collected in three blocks: (1) baseline resting EEG for 5 minutes, (2) active or sham stimulation, (3) post-stimulation resting EEG for 5 minutes. For the resting EEG, the participants were instructed to close their eyes for 5 minutes. During the stimulation, they performed the task described above.

EEG data were preprocessed using the EEGLAB toolbox (53). EEG data were down-sampled to 500 Hz and bandpass filtered (0.5–100 Hz). The cleanLine plugin of EEGLAB was used to remove the 50 Hz electrical line noise. Channels containing excessive noise or artifacts were identified and removed using the EEGLAB plug-in FASTER (54). Across all participants, on average 2.10 (SD = 1.09, range 0–5) channels per subject were removed. Independent component analysis (ICA) was performed on continuous EEG data to remove ocular artifacts and generic noise. Artifactual independent components (ICs) were identified and removed from the data using the ICLabel plug‐in (55) of EEGLAB and through visual inspection of individual ICs. On average, 13.2 ICs (SD = 5.62, range 7-29) per subject were removed from the data. EEG data were then segmented into 1‐s epochs. Epochs containing artifacts were removed using a voltage threshold (± 100μV) rejection. This procedure removed on average 21.2 (SD = 40.3) epochs per subject. After artifact rejection, any missing channels were interpolated using spherical interpolation. The epoched data were then re-referenced to the average of all channels.

### 2.6 Spectral power analysis

The EEG epochs were processed using Welch’s method to estimate the PSD for each channel. Specifically, we applied a Hanning window of 0.5 seconds with a 0.25-second overlap to each epoch. The resulting PSD values (µV²/Hz) were averaged across all epochs for each channel and log-transformed (log10). To avoid negative values, 1 was added to the power values before the log transformation.

Power was extracted at 5 Hz and for the theta (4–6 Hz), delta (1–3 Hz), and alpha (7–12 Hz) bands. Finally, power was averaged across a cluster of electrodes (FC1, FC3, F5, Fz) surrounding the stimulation site (F3) in each frequency band of interest. Since the FC1, FC3, F5, and Fz electrodes surround the stimulation site (F3), analyzing power across this cluster allows for a more comprehensive assessment of the effects of stimulation on the targeted region. Although the primary frequency of interest was 5 Hz, we also analyzed power in the delta, theta, and alpha bands to assess whether the power modulation was specific to the stimulation frequency.

### 2.7 Statistical analyses procedure

We recorded response accuracy and reaction time for behavioral data analysis. We used d-prime values as outcome measures for response accuracy. D-prime, a discriminability index adapted from signal detection theory (56), measures the ability to correctly identify targets while minimizing false alarms and has been shown to have high sensitivity to detecting true signals or targets (57). Participants’ responses were categorized as either hits, misses, false alarms, or correct rejections. These response types were used to calculate d-prime metrics. Hit rate (H) was calculated as the proportion of correctly identified targets (i.e., H = Hits/Hits+Misses), and false alarm rate (FA) was the proportion of incorrect identifications (FA = False Alarms/False Alarms+Correct Rejections). To avoid issues with extreme values (e.g., hit rates of 1.0 or false alarm rates of 0), values were adjusted following standard practices: if H or FA equaled 1.0 or 0, they were adjusted by 1/2N​, where N represented the number of trials. The d-prime index was then computed as the difference between the z-transformed hit rate and false alarm rate (d’ = Z(H)−Z(FA)). A repeated measures ANOVA was conducted with factors of Stimulation (Sham, tACS) and Task-load (Low, High) on d-prime scores. Reaction time (RT) was defined as the interval between the probe stimulus and the button press. RTs were also calculated for the 4 categories of responses. We performed a repeated measures ANOVA with factors of Stimulation (Sham, tACS), Task-load (Low, High), and Response (Hit, Miss, False Alarm, Correct Rejection). Baseline and post-stimulation EEG spectral power were compared using a repeated measures ANOVA with factors of Stimulation (Sham, tACS) and Time (Baseline, Post-stimulation). ANOVA was performed separately for each measured frequency. Follow-up analyses were performed to compare baseline and post-stimulation EEG spectral power using paired two-tailed t-tests. Power and behavioral data were normally distributed according to Shapiro-Wilk tests. Throughout the analyses, extreme scores (values exceeding ±3 SD from the mean) were excluded as outliers. Statistical analyses were performed using R.

## 3 Results

### 3.1 D-prime

**Figure 2:**
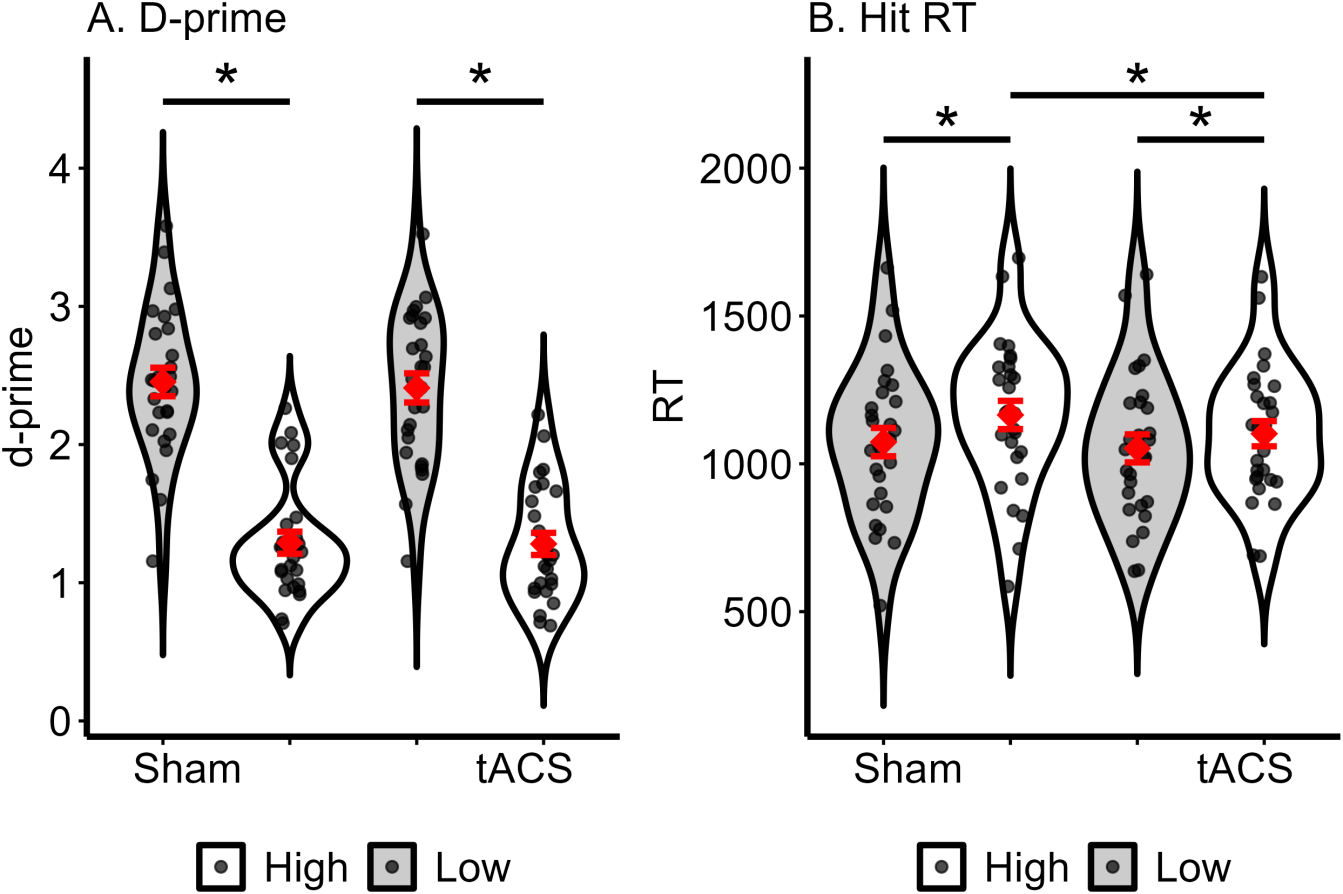
Effects of Sham and tACS stimulations on working memory performance, measured by d-prime **(A)** and reaction time (RT) for hit responses **(B)**. The violin plots display the distribution of individual data points shown as jittered dots. Red diamonds mark the mean and red error bars show the standard error. Significant differences between specific conditions are denoted by asterisks above the plots. * *p* <.05.

**Table 1** presents the means and standard deviations of the d-prime score. The ANOVA with factors Stimulation (Sham, Theta) and Task-load (Low, High) on d-prime scores revealed a main effect of Task-load (*F*(1, 26) = 131.66, *p* <.001, *η^2p^* = .835). *Post hoc* comparison with Bonferroni corrections showed that performance was significantly higher in low-load trials compared to high-load trials in both Sham (*T*(26) = 11.31, *p* <.001) and tACS (*T*(26) = 8.90, *p* <.001) conditions. Neither the main effect of Stimulation (*F*(1, 26) = .13, *p* = .725, *η^2p^* = .005) nor the Stimulation x Task-load interaction (*F*(1, 26) = .09, *p* = .769, *η^2p^* = .003) was significant. The results suggest that participants had higher accuracy in low-load trials and made more errors in high-load trials regardless of stimulation conditions (**Figure 2A**).

**Table 1:** Means and standard deviations (in parentheses) of d-prime scores and reaction times (ms) for correct responses in low- and high-load trials for Sham and tACS conditions.

	Low Load	High Load
d-prime		
Sham	2.41 (.57)	1.28 (.41)
tACS	2.41 (.55)	1.27 (.42)
Hit RT		
Sham	1074 (254)	1165 (253)
tACS	1052 (251)	1102 (226)

### 3.2 Reaction time

The three-way repeated-measures ANOVA of reaction time revealed the main effect of Task-load (*F*(1, 27) = 41.13, *p* <.001, *η^2p^* = .604), resulting from overall faster response for low load trials compared to the high load trials. The main effect of Response was also significant (*F*(3, 81) = 4.71, *p* = .004, *η^2p^* = .148). The main effect of Stimulation was not significant (*F*(1, 27) = 1.73, *p* = .199, *η^2p^* = .060). However, the Stimulation x Task-load x Response interaction was significant (*F*(3, 81) = 4.31, *p* = .007, *η^2p^* = .138). To explore the interaction, follow-up Stimulation x Task-load ANOVA was performed within each response category. The ANOVA for Hit response revealed a main effect of Stimulation (*F*(1, 27) = 4.93, *p* = .035, *η^2p^* = .154) and Task-load (*F*(1, 27) = 30.82, *p* <.001, *η^2p^* = .533). The Stimulation x Task-load interaction was also significant (*F*(1, 27) = 4.52, *p* = .043, *η^2p^* = .143). *Post hoc* comparisons after the Bonferroni correction showed that participants made significantly faster hit responses in high-load trials during active tACS than Sham stimulation (*T*(27) = 3.58, *p* = .008) (**Figure 2B**). In contrast, there was no significant difference in RTs for hit responses in low-load trials between Sham and active tACS stimulation conditions (*T*(27) = .86, *p* = .395) (**Figure 2B**). **Table 1** presents a summary of RTs for hit responses. The ANOVA for Miss response revealed a main effect of Task-load (*F*(1, 27) = 11.64, *p* = .002, *η^2p^* = .301), resulting from faster overall RT in Low-load (*M* = 1027) compared to High-load (*M* = 1101) trials across stimulations. The main effect of Stimulation (*F*(1, 27) = .81, *p* = .375, *η^2p^* = .029) and Stimulation x Task-load interaction (*F*(1, 27) = .37, *p* = .55, *η^2p^* = .013) were not significant. The ANOVA for False alarms response revealed a main effect of Task-load (*F*(1, 27) = 10.26, *p* = .003, *η^2p^* = .277), resulting from higher overall RT in High-load (*M* = 1207) compared to Low-load (*M* = 937) trials across stimulations. The main effect of Stimulation (*F*(1, 27) = .77, *p* = .388, *η^2p^* = .028) and Stimulation x Task-load interaction (*F*(1, 27) = .003, *p* = .953, *η^2p^* = .00) were not significant. The ANOVA for Correct rejections response revealed a main effect of Task-load (*F*(1, 27) = 63.53, *p* <.001, *η^2p^* = .702), resulting from higher overall RT in High-load (*M* = 990) compared to Low-load (*M* = 878) trials across stimulations. The main effect of Stimulation (*F*(1, 27) = .44, *p* = .51, *η^2p^* = .016) and Stimulation x Task-load interaction (*F*(1, 27) = .69, *p* = .414, *η^2p^* = .025) were not significant. In summary, the RT analyses revealed that participants responded faster overall in low-load trials, and tACS stimulation specifically accelerated responses when participants correctly recognized stimuli presented during the encoding phase in the high-load trials. However, tACS did not affect error response times and the RTs related to the correct rejection of the stimuli. These behavioral results suggest that tACS may enhance the speed of accurate responses, particularly in high-load trials. However, the results should be interpreted cautiously rather than as conclusive evidence of a load-specific effect. Instead, they point to a promising trend that warrants further investigation, with additional data needed to confirm the reliability and replicability of the observed effects.

### 3.3 Spectral power

**Figure 3:**
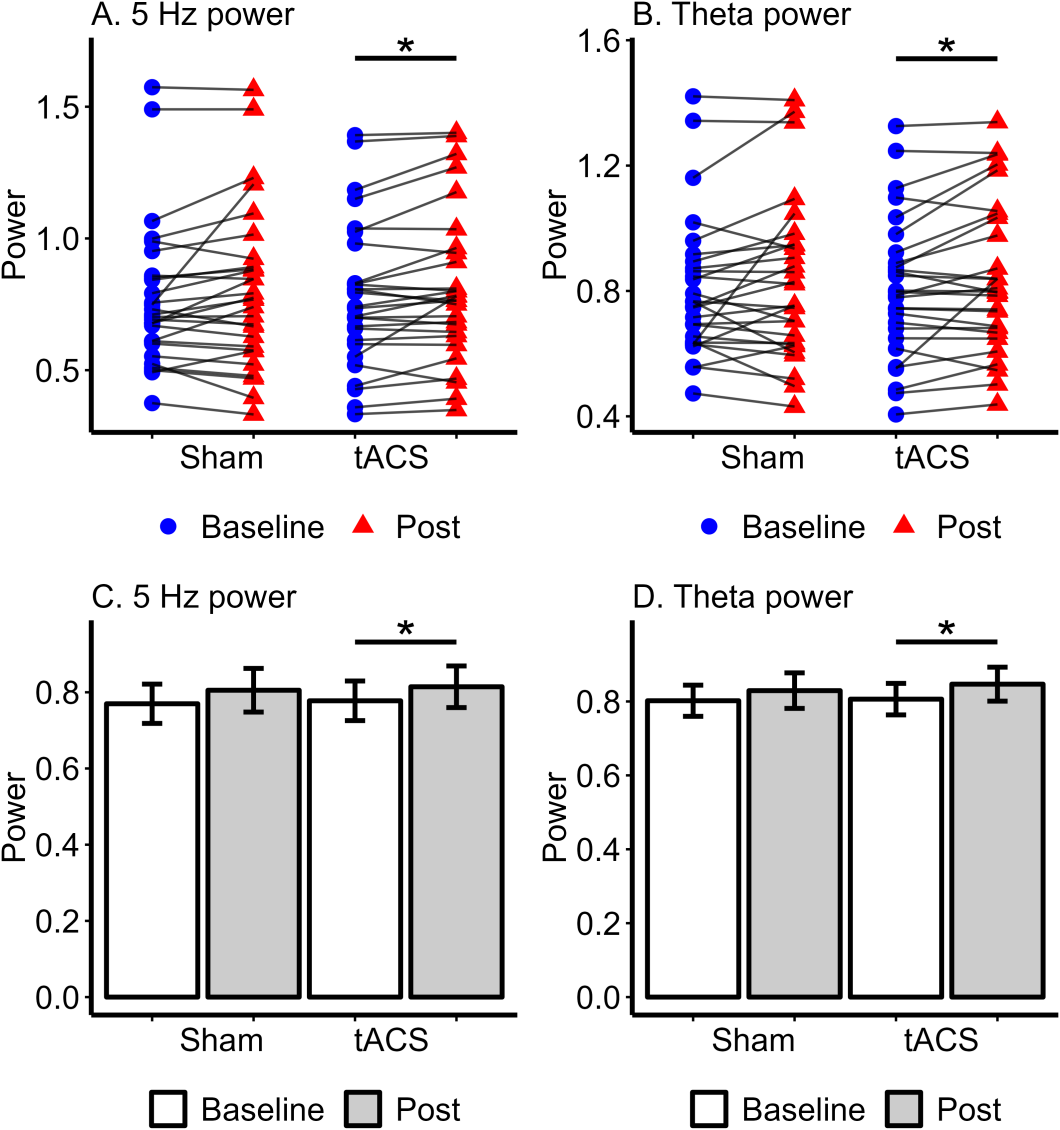
Spectral power at 5 Hz and theta band in Sham and tACS stimulation conditions. The upper **(A, B)** show individual power values with lines connecting baseline (pre-stimulation) and post-stimulation powers for each participant. **(A)** represents 5 Hz power, while **(B)** shows Theta power, with statistical significance indicated by an asterisk (*) between the baseline and post-stimulation powers in the tACS condition. The lower **(C, D)** present bar plots of mean power with standard error for each condition. **(C)** shows 5 Hz power, and **(D)** shows Theta power, with an asterisk (*) highlighting significant differences between baseline and post-tACS power. * p <.05.

For power at the stimulation frequency of 5 Hz, the ANOVA revealed a main effect of Time (*F*(1, 27) = 7.15, *p* = .013, *η^2p^* = .210). Neither the main effect of Stimulation (*F*(1, 27) = .13, *p* = .723, *η^2p^* = .005) nor the Stimulation x Time interaction (F(1, 27) = .003, *p* = .952, *η^2p^* = .00) was significant. We further explored the main effect of Time by comparing baseline and post-stimulation power. Based on our *a priori* hypotheses, we conducted paired t-tests within each condition to investigate potential condition-specific effects. In the active tACS condition, post-stimulation power (*M* = .814, *SD* = .29) was significantly higher than baseline power (*M* = .778, *SD* = .28) at the stimulation frequency of 5 Hz (*T*(27) = 2.78, *p* = .01, Cohen’s *d* = .526). In contrast, no significant difference was observed between baseline (*M* = .805, *SD* = .30) and post-stimulation (*M* = .770, *SD* = .27) powers in the sham condition (*T*(27) = 1.71, *p* = .098, Cohen’s *d* = .324). The ANOVA comparing baseline and post-stimulation theta band power in sham and tACS stimulation conditions also revealed a main effect of Time (*F*(1, 27) = 5.17, *p* = .031, *η^2p^* = .160). The main effect of Stimulation (*F*(1, 27) = .28, *p* = .601, *η^2p^* = .01) and Stimulation x Time interaction (*F*(1, 27) = .306, *p* = .585, *η^2p^* = .011) were not significant. In the active tACS condition, post-stimulation (*M* = .847, *SD* = .24) theta power was significantly higher than baseline (*M* = .806, *SD* = .23) theta power (*T*(27) = 2.49, *p* = .019, Cohen’s *d* = .470). However, theta power did not differ between baseline (*M* = .802, *SD* = .22) and post-stimulation (*M* = .829, *SD* = .26) in the sham condition (*T*(27) = 1.26, *p* = .218, Cohen’s *d* = .239). Comparisons of baseline and post-stimulation power did not show significant differences in the delta and alpha bands for either the active tACS or sham conditions (all *p* >.05). Spectral power analyses demonstrated that 5 Hz tACS induced a significant increase in power and the power modulation was specific to the stimulation frequency (**Figure 3**).

## 4 Discussion

This study investigated how tACS at a slow theta frequency (5 Hz) modulated brain rhythms and task performance. Sham or 5 Hz tACS was applied to the left DLPFC while participants performed WM tasks with varying cognitive loads. RT and accuracy were measured during both tACS and sham conditions to assess task performance. Resting state EEG was collected before and after the stimulations, and spectral power was computed from EEG data. The results showed that tACS significantly enhanced task performance, particularly in high-load trials, as demonstrated by faster reaction times. Additionally, there was a significant increase in post-tACS spectral power at 5 Hz and in the theta band. This study builds on our previous work (33), in which we demonstrated that tACS at a slow theta frequency (5 Hz) over the left DLPFC improved working memory performance under high cognitive load conditions. In the present study, we found selective enhancement of RT in WM tasks. Additionally, we extend our earlier research by showing a significant increase in post-tACS spectral power, indicative of after-effects.

Our findings demonstrated that tACS improved RT in WM tasks selectively for cognitively demanding tasks. Specifically, response speed in high-load trials was significantly faster in the tACS condition compared to the sham condition. Notably, there were no effects of any stimulation on accuracy, suggesting that the improved response speed in high-load trials in the tACS condition did not involve a speed-accuracy trade-off. In a previous study, we found that 5 Hz tACS preferentially enhanced performance on high WM load tasks (33). Furthermore, our results are consistent with numerous previous studies reporting that tACS administered in the alpha (25, 36), theta (19, 33), and gamma (58, 59) ranges positively impacted cognitive functions. Hoy et al. (58) found that gamma-tACS improved WM performance only for stimuli that required greater cognitive load. The selective improvement of cognitive functions following tACS has also been reported in other domains (36, 59). The selective improvement in WM performance induced by tACS may result from the enhanced synchronization of neural oscillations. tACS is believed to entrain endogenous oscillations by enhancing coherence and synchronization of neuronal oscillations within and across brain regions (60, 61). The improved synchronization facilitates more efficient neural communication, which is particularly beneficial for cognitively demanding tasks in which the need for coordinated neural activity is higher. Furthermore, neural synchronization is considered pivotal in representing information in WM (62–64). The complex cognitive processes underlying WM are mediated by oscillatory activity across multiple frequency bands, involving both independent contributions and cross-frequency coupling (16–18, 21, 22). However, evidence suggests that specific frequency bands may play a more critical role in certain aspects of WM. Notably, frontal theta power is associated with memory load, with increased power correlating with the number of items maintained (65, 66). These findings highlight the facilitatory role of increased theta power in WM performance under higher cognitive load. Moreover, the absence of effects in the low-load condition is not surprising, given that low cognitive demand often leads to high performance and may cause ceiling effects. Therefore, the mechanism of tACS in enhancing neural synchrony likely accounts for the selective improvement of performance in WM tasks observed in this study. Our results illustrate the involvement of DLFPC in visual WM and the importance of theta oscillations in WM processes.

We found that 5 Hz tACS over the left DLPFC significantly increased spectral power post-stimulation compared to the pre-stimulation baseline level. The power enhancement, known as after-effects, was limited to the theta band and did not occur in the adjacent delta and alpha frequency bands. Our findings are consistent with the idea that tACS modulates brain oscillations in the frequency band corresponding to the stimulation frequency (25, 29, 30, 50). Although the exact neuronal mechanism of tACS is not fully understood, current research suggests that the after-effects may be primarily driven by neuronal entrainment or synaptic plasticity (26, 67, 68). The neuronal entrainment theory suggests that continuous stimulation with an oscillating current causes more individual neurons to synchronize their activity with the external rhythm, leading to a power increase across the entire network (68). It further suggests that neurons oscillating within the stimulation frequency range will become synchronized and entrained, while those with intrinsic frequencies outside this range will remain unaffected (68). Our findings may be explained by neuronal entrainment, as elevated power was observed specifically in the theta band while power in surrounding frequency bands did not change.

### 4.1 Limitations

Although this study was carefully designed and conducted, some limitations should be noted. The placement of the stimulation electrodes was based on previous studies, which may not be ideal. Given that tACS is more localized, individual differences in head anatomy could affect the results. Future studies using imaging techniques could address this issue. Additionally, we were unable to analyze the EEG data recorded during tACS due to artifacts introduced by the electrical stimulation. Currently, there is no established method to eliminate this noise effectively. Overcoming this challenge would provide crucial insights into the underlying processes of tACS and its effects on brain function. Our evaluation of working memory performance was based solely on reaction time (RT), accuracy and spectral power in the theta band. While these metrics are commonly used to assess task performance and neural activity, working memory is influenced by a range of factors, including cognitive strategies and individual differences in processing speed. Therefore, relying exclusively on RT and spectral power may not capture the full complexity of the cognitive processes involved in working memory tasks. Finally, we did not observe any effects of tACS on accuracy. While this outcome was not anticipated, previous research indicated that tACS may selectively improve task performance.

### 4.2 Conclusion

Our findings demonstrated that the application of tACS at a slow theta frequency (5 Hz) over left DLPFC improved performance in WM tasks preferentially in the higher cognitive load condition. Furthermore, the results showed a significant increase in post-tACS spectral power, known as after-effects. Our findings highlight the potential of tACS as a non-invasive brain stimulation method for modulating brain activity and enhancing cognitive function. Given that cognitive deficits are common in psychiatric and neurological conditions, these findings have significant implications for the clinical application of tACS in enhancing cognitive functions and overall wellbeing in clinical populations.

## Data Availability

The raw data supporting the conclusions of this article will be made available by the authors, without undue reservation.
